# Augmented reality near-eye display using Pancharatnam-Berry phase lenses

**DOI:** 10.1038/s41598-019-42979-0

**Published:** 2019-04-29

**Authors:** Seokil Moon, Chang-Kun Lee, Seung-Woo Nam, Changwon Jang, Gun-Yeal Lee, Wontaek Seo, Geeyoung Sung, Hong-Seok Lee, Byoungho Lee

**Affiliations:** 1 School of Electrical and Computer Engineering and Inter-University Semiconductor Research Center, Seoul National University, Gwanak-Gu Gwanakro 1, Seoul, 08826 Republic of Korea; 2 Imaging Device Lab, Samsung Advanced Institute of Technology, Samsung Electronics, Suwon, Gyeonggi-do Republic of Korea

**Keywords:** Imaging and sensing, Electronics, photonics and device physics

## Abstract

An augmented reality (AR) near-eye display using Pancharatnam-Berry (PB) phase lenses is proposed. PB phase lenses provide different optical effects depending on the polarization state of the incident light. By exploiting this characteristic, it is possible to manufacture an AR combiner with a small form factor and a large numerical aperture value. The AR combiner adopted in the proposed system operates as a convex lens for right-handed circularly polarized light and operates as transparent glass for left-handed circularly polarized light. By merging this combiner with a transparent screen, such as diffuser-holographic optical elements (DHOEs), it is possible to make an AR near-eye display with a small form factor and a wide field of view. In addition, the proposed AR system compensates the chromatic aberration that occurs in PB phase lens by adopting three-layered DHOEs. The operating principle of the proposed system is covered, and its feasibility is verified with experiments and analysis.

## Introduction

Augmented reality (AR) near-eye displays have been in the limelight for the past few years. Since these devices can effectively deliver virtual worlds to observers, recent studies on AR fields have been focused on developing various types of near-eye displays. The most widely known method is the use of prisms, such as beam splitters or freeform optics. Beginning with Google Glass in 2013, numerous kinds of AR near-eye displays using prisms have been developed. For instance, Meta Company released an AR display named Meta 2 to the commercial market^1^. This device provides a 90-degree field of view (FOV) using a freeform half-mirror. IN addition to the commercialized products, many AR technologies using prisms have been developed in academia, such as multifocal displays or off-axis see-through head-mounted displays^2–5^. The use of prisms in AR near-eye displays has the advantage of providing high transparency meaning that the observer can perceive a relatively clear real world scene. However, to provide a wide FOV in these systems, it is difficult to reduce the overall system form factor.

To overcome this trade-off relationship, various types of AR combiners are suggested. Holographic optical elements (HOEs) and diffractive optical elements (DOEs) are some of these combiners. HOEs and DOEs have received a great attention due to the angular and wavelength selectivity^6^. These devices react as optical elements only for the light with specific incident angle and wavelength while they behave like a transparent glass for the rest of the light. Exploiting these characteristics, various types of light field-based near-eye AR displays and holographic near-eye AR displays have been proposed^7–13^. In 2016, the first version of Microsoft Hololens was released in the commercial market^14^. This product, which adopts DOEs and waveguides, sparked great interest. Although there are some shortcomings, such as insufficient FOV and relatively low image resolution, the release of Hololens was an important event that took the commercialization of AR displays one step forward. Employing HOEs and DOEs as AR combiners, however, has several limitations. If HOE or DOE lenses are used as imaging optics, severe astigmatism occurs^15^. To avoid this issue, HOEs or DOEs have been applied to provide a Maxwellian view or holography in several studies, but these systems require additional cost to achieve a sufficient eye box^16,17^. There have been studies related to AR combiners using polarization characteristics to achieve high numerical aperture (NA), such as an anisotropic lens or a metasurface lens^18,19^. These systems represent full-color virtual images with a sufficient FOV by adopting the polarization multiplexing method. However, the manufacturing process of these methods are tricky and expensive. Additionally, these approaches are difficult to commercialized due to the limitation in mass production.

In this paper, an AR near-eye display with a combiner that consists of stacked Pancharatnam-Berry (PB) phase lenses is proposed. The most remarkable characteristic of the AR combiner is that it represents different optical effects depending on the polarization state of the incident light. If light with right-handed circular (RHC) polarization is use for illumination, the combiner is activated as a convex lens while light with left-handed circular (LHC) polarization passes through the combiner without undergoing a phase shift. In other words, as the light from the display device is projected with an RHC polarization state, the observer can perceive a floating virtual image. At the same time, if the light from the real world scene is set to an LHC polarization state, the scene is delivered to the observer without any distortion. The second strength of the proposed combiner is a high NA. The focal length and the radius of the combiner are 22.5 mm and 25.4 mm, respectively. Due to the polarization characteristic of the combiner, it is possible to place the lens directly in front of the observer’s eye. Thus, the overall system can present a relatively wide field of view with a sufficiently large eye box. Lastly, the proposed combiner is light and small. Unlike general commercialized lenses, the PB phase lens is constructed by coating liquid crystal molecules on the flat surface, and therefore, the thickness of the PB phase lens is smaller than 500 µm. Since the proposed AR combiner consists of two-layer PB phase lenses, the thickness of the combiner does not exceed 1 mm.

The contribution of our work is the proposal of the AR near-eye display using the combiner composed of PB phase lenses. We combine the AR combiner with three-layer diffuser HOEs (DHOEs) to achieve AR with a proper eye box and deal with the chromatic aberration simultaneously. The proposed system achieved a compelling FOV of 80 degrees. A detailed explanation of the system and experimental results are covered in the following sections.

## Results

### Augmented reality combiner using Pancharatnam-Berry phase lenses

PB phase refers to the phase shift gained by electromagnetic waves that go through the continuous sequence of polarization state transformations following a closed path in a Poincare sphere^20^. Different from conventional phase or amplitude gratings, PB optical elements operate by locally modifying the polarization state of light waves passing through them. The properties of PB optical elements have been used for wide FOV nonmechanical beam steering applications^19,20^. One of the most interesting applications for PB optical elements is a PB phase lens that modulates the phase using liquid crystal (LC) molecules to provide a special profile depending on the polarization state of the input light. Recently, many studies have been performed on microscopic imaging fields and AR display techniques using PB phase lens^21,22^.

**Figure 1:**
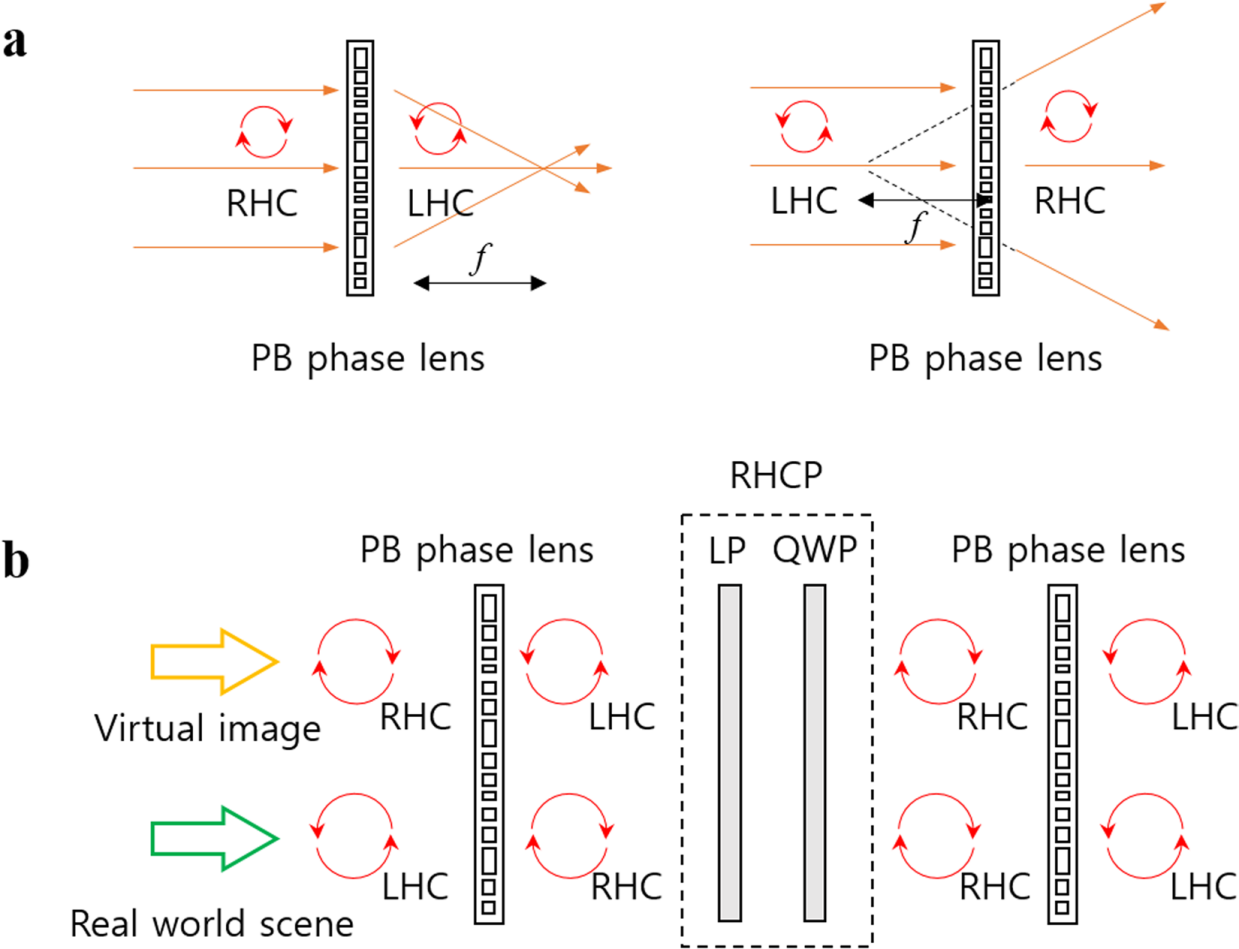
Schematic diagram of the PB phase lens and the proposed strategy. (**a**) The operation principle of the general PB phase lens and (**b**) schematic diagram of PBLIC.

An AR combiner consisting of stacked commercialized LC-based PB phase lenses was presented at a conference as a previous work for this research^23^. We named the combiner PBLIC (Pancharatnam-Berry phase lens-image combiner) for convenience. This work proposes a compact AR system scheme using PBLIC. Figure 1(a) represents the operation principle of the general PB phase lens adopted in the system^24^. When the light with RHC polarization state is illuminated, the PB phase lens is activated as a convex lens and the output light is modulated to have an LHC polarization state. The PB phase lens operates as a concave lens for LHC polarized input light, and the output light is modulated to an RHC polarization state.

Figure 1(b) presents a schematic diagram of PBLIC. PBLIC consists of three layers: two PB phase lenses and one RHC polarizer (RHCP) between the PB lenses. As shown in the figure, if the system is illuminated by a beam with RHC polarization, the beam passes through two identical convex lenses with focal length *f*. Since the thickness of each component is thin enough, the overall focal length of the combiner for the incident RHC polarized beam is approximately *f*/2. If the incident beam has LHC polarization, the beam passes through a concave lens with focal length *−f* in the first layer. Then, the polarization state is modulated to RHC polarization after the beam passes through the RHCP. In the last layer, the beam passes through a concave lens with focal length *f*. Thus, the effective focal length for the incident LHC polarized beam is infinity, which means the beam is delivered without experiencing any optical effects. In the system, the virtual image and the real world scene are provided with an RHC polarization state and an LHC polarization state, respectively. The brightness of the virtual image and the real world scene is reduced by 50% while the images pass through PBLIC due to the RHCP, which consists of one linear polarizer (LP) and one quarter wave plate (QWP).

The largest issue with PBLIC is severe chromatic aberration. The phase modulations for two different light waves passing through the PB phase lens should be equal, as shown below^19,25^:

1 $$\frac{2\pi }{{\lambda }_{1}}(\sqrt{{z}_{1}^{2}+{(x-{x}_{1})}^{2}+{(y-{y}_{1})}^{2}}-{z}_{1})=\frac{2\pi }{{\lambda }_{2}}(\sqrt{{z}_{2}^{2}+{(x-{x}_{2})}^{2}+{(y-{y}_{2})}^{2}}-{z}_{2}),$$

where *λ*_*n*_ is the wavelength, (*x*, *y*) is the coordinates on the metasurface, and (*x*_*i*_, *y*_*i*_, *z*_*i*_) represents the location of a particular point in the 3D space at wavelength *λ*_*i*_. If the parallel light is illuminated, (*x*_1_, *y*_1_, *λ*_1_*z*_1_) should be equal to (*x*_2_, *y*_2_, *λ*_2_*z*_2_) under the paraxial approximation to satisfy the above equation. Thus, *λ*_1_ *f*_1_ is approximately equal to *λ*_2_ *f*_2_. In other words, when the parallel polychromatic wave with an RHC or LHC polarization state passes through the PB phase lens, a number of focal spots are generated. In addition, the distance between the PB phase lens and the focal spot is inversely proportional to the wavelength of each wave component. In the case of PBLIC, an input wave with an RHC polarization state undergoes the above phenomenon twice, meaning that the chromatic aberration of the virtual image occurs twice. On the other hand, the LHC polarized input wave undergoes two conjugate phenomena, as shown in Fig. 1(b). Thus, the real world scene is delivered clearly without the chromatic aberration. In fact, this is another advantage of PBLIC since it is possible to resolve the chromatic aberration in virtual images systemically, but it is difficult to compensate for the chromatic aberration in a real world scene because it is delivered to the observer without additional computational processes.

### Augmented reality system using PBLIC and compensation for the chromatic aberration

In this section, the overall AR near-eye display system is presented. As mentioned above, the main issue to deal with in PBLIC is chromatic aberration. To compensate for this issue systemically, the exact focal lengths of PBLIC for three wavelengths of light sources using a laser projector (Celluon, Picopro wavelength 639 nm, 522 nm, 445 nm) are measured. The measurement setup is very simple. A collimated beam from the projector is used to illuminate the PBLIC, and a CCD sensor on the linear stage detects the focal spots of the three light sources. Then, the distance between the PBLIC and the CCD sensor is measured. The measured focal lengths for the red, green and blue sources are 18.65 mm, 22.29 mm, and 25.21 mm, respectively. In our design, the virtual image was floated 2 m away from the PBLIC. Under these conditions, the gaps between the PBLIC and screens should be 18.53 mm, 22.1 mm, and 25 mm for red, green and blue, respectively, as given by the thin-lens formula.

There are several requirements for the screens to be adopted in the proposed AR system. First, the screens must be transparent to external light while they diffuse the light from the display device. Second, each of the screens should react only to the light with specific wavelength. Since the gaps between each screen are very narrow, it is difficult to convey the image without passing through the front layer. Thus, the first-layer screen should react only to the red light source. Likewise, the second- and the third-layer screens should diffuse only the green and blue light sources, respectively. These two conditions agree with the characteristics of holographic optical elements (HOEs). HOEs are thin volume gratings recorded inside the photopolymer. HOEs react only for light with a specific incident angle and wavelength while behaving like a transparent glass for the rest of the light. Owing to this characteristic, diffuser-HOEs (DHOEs) are suitable to be adopted in the system as the transparent screens. However, there is a limitation in conventional DHOEs.

**Figure 2:**
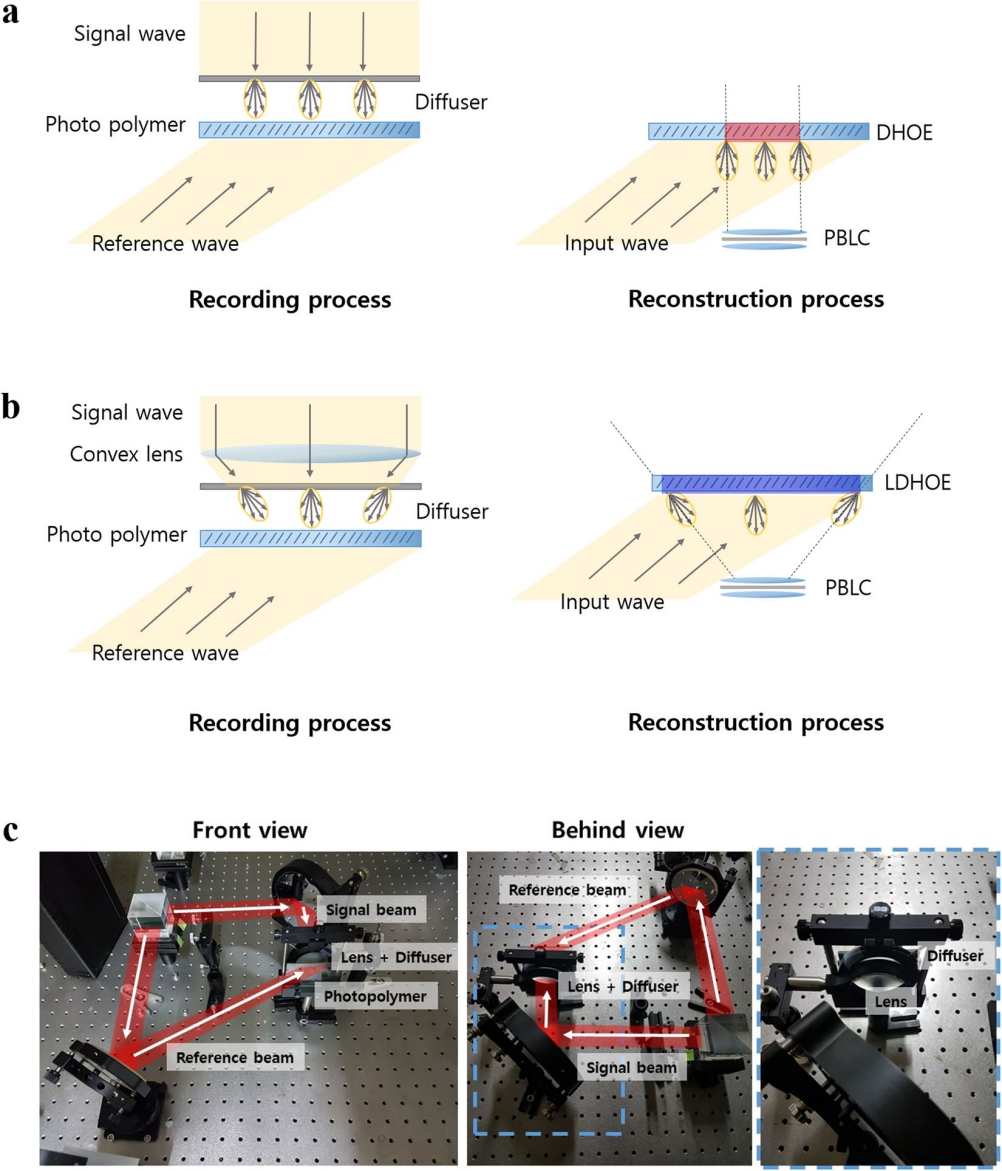
DHOE and LDHOE recording and reconstruction process. (**a**) Recording and reconstruction process of conventional DHOE, (**b**) LDHOE, and (**c**) the recording setup of LDHOE.

Figure 2(a) shows the recording and reconstruction processes of conventional DHOEs. As shown in the figure, when a DHOE diffuses light at a recorded diffusing angle, only the image represented in the red region can be conveyed to the observer through the combiner. Light from outside of the red region is partially delivered or not delivered at all. This phenomenon, also called the “vignetting effect”, makes the observer unable to perceive the entire image presented in the screen, and the observer regards this situation as having a narrow FOV. If the recorded diffusing angle is very large, this problem can be reduced, but in the case of DHOE, it is difficult to record the diffuser with a large diffusing angle because it adversely affects both the efficiency and transparency of the DHOE. To deal with this problem effectively, we suggest a new type of DHOE named lens-DHOE (LDHOE). Figure 2(b) represents the recording and reconstruction process of LDHOE. The LDHOE recording process is constructed by adding a convex lens in front of the diffuser in the conventional DHOE recording process. Due to the convex lens, the direction vectors of the chief rays converge after they pass through the diffuser. As the direction of the chief rays is tilted, the area of the screen that passes through the aperture becomes wider as the blue region. Additionally, the rays converge once more due to the AR combiner, and the FOV ultimately becomes wider than the system with conventional DHOE. Figure 2(c) represents the LDHOE recording setup. The incident laser beam splits into two paths. One beam is used as a signal beam that passes through the convex lens and diffuser, while the other beam is used as a reference beam. The diffusing angle of the adopted diffuser is 10 degrees and the focal length of the additional lens is 200 mm, 100 mm, and 80 mm for the red, green, and blue channels, respectively. The detailed recording process and the reason for choosing these focal lengths are provided in the method section.

### Experimental results

**Figure 3:**
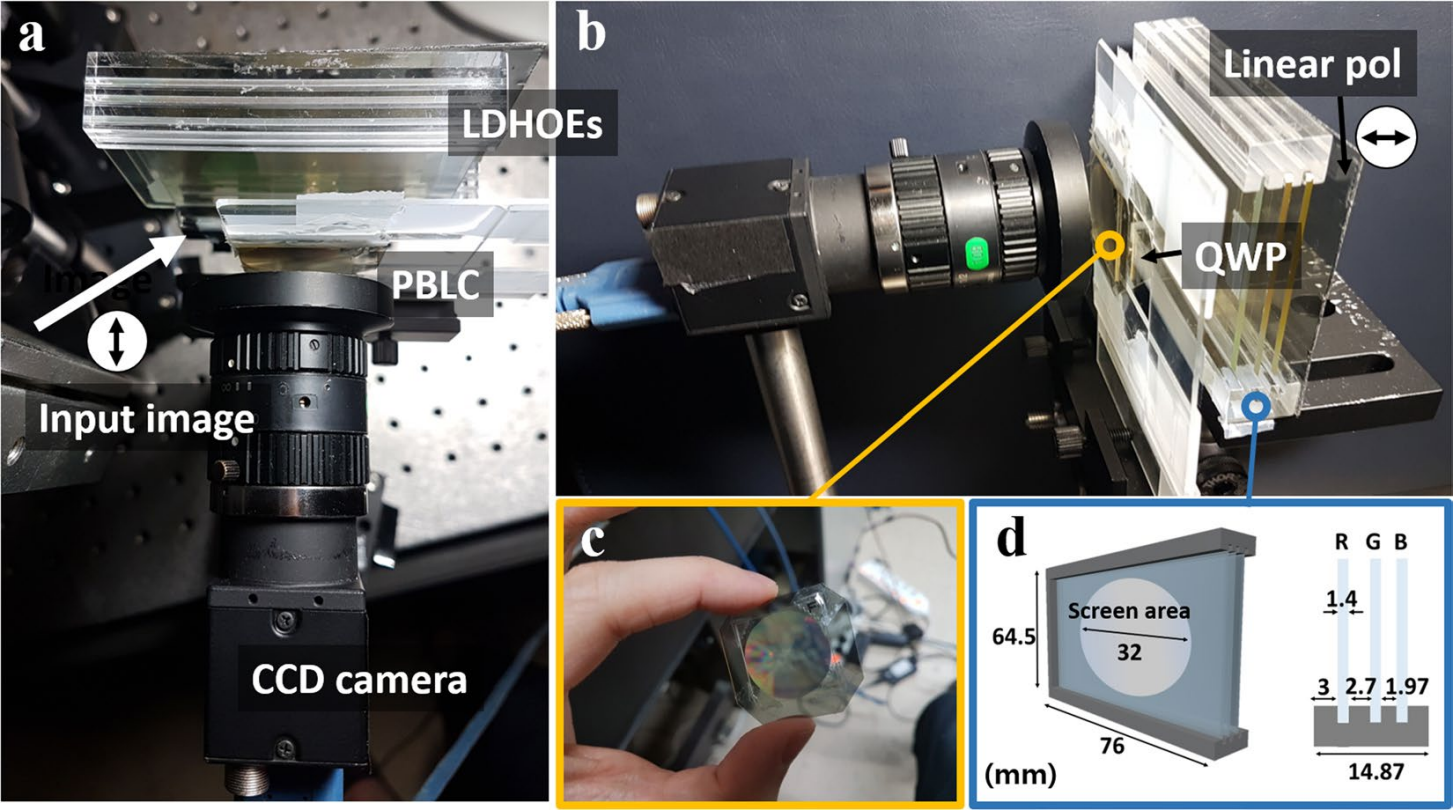
Experimental setup of the proposed AR near-eye display system using a PBLIC. (**a**) Top view of the overall setup, (**b**) side view of the overall setup, (**c**) PBLIC, and (**d**) the schematic diagram of the stacked LDHOEs and numerical values of the screen size and gaps between each LDHOE.

Figure 3 shows the experimental setup. Three LDHOEs are stacked in the acrylic jig. As mentioned in the previous section, a red LDHOE is placed in the front. Green and blue LDHOEs are placed in sequence with an appropriate gap. In front of the layered LDHOEs, the PBLIC is placed, and the image is projected from the laser projector with an incidence angle of 33 degrees. The beam from the projector is collimated by a 2-inch convex lens with a 75 mm focal length. Two linear polarizers and one quarter wave plate (QWP) are adopted in the system to adjust the polarization states of the lights from the projector and the real world scene. One linear polarizer is attached behind the LDHOEs, and the other is placed in front of the laser projector. These two linear polarizers are orthogonally aligned. Finally, a QWP is located between the PBLIC and LDHOEs to modulate the polarization states. Even though the combination of a linear polarizer and a QWP has the same function as circular polarizers, the system cannot adopt circular polarizers due to the characteristic of HOEs. For the output light from the HOEs, the degree of conservation of polarization state is different for the TE and TM polarization states^26^. Thus, if the circularly polarized beam is illuminated to HOEs, the polarization state of the output beam would be elliptically polarized. Since the PBLIC reacts to the light with an exact circular polarization state, critical noise occurs if the input beam has an elliptical polarization state. By employing linear polarizers and QWP in the system, the virtual image and the real-world scene are delivered with RHC and LHC polarization states, respectively.

**Figure 4:**
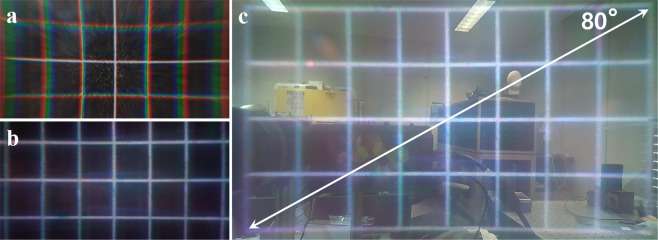
The virtual images of a white grid floated by the PBLIC. (**a**) The virtual image of the white grid printed on a sheet of paper. (**b**) The virtual image of the grid image presented by the proposed system after the entire compensation step. (**c**) AR scene of the white grid with an FOV of 80 degrees.

Due to some distortions, several image compensation steps are necessary to present intact virtual scenes. First, a keystone distortion occurs due to oblique projection from the laser projector. Second, a pincushion distortion occurs from the PBLIC since it acts as a convex lens for the input image from the projector. Last, the color uniformity should be modified. Since the recording process is not perfect, each part of the LDHOE has slightly different efficiency, and each LDHOE shows different efficiency. The keystone distortion is compensated for by using the planar homography method. A transformation matrix can be derived by comparing coordinate values of four corner points between the target image and the distorted image. Using the inverse matrix of this transformation matrix, the keystone distortion can be resolved^27^. Pincushion distortion can be compensated for by various methods. In this work, the degree of pincushion distortion is calculated by adopting the checkerboard image-method. Then, the compensation model for the radially symmetric distortion is applied^28^. To settle the color uniformity issue, red, green, and blue square images are captured, and gamma values for every pixel are normalized. Figure 4(a,b) represent the virtual images of a white grid floated by the PBLIC. In Fig. 4(a), a white grid is printed on a sheet of paper, while in Fig. 4(b), the grid is captured from the proposed system after the entire compensation step. As shown in these figures, a severe chromatic aberration occurs from the PBLIC if a single display panel is adopted, and this aberration can be resolved by the proposed method. Figure 4(c) presents the AR scene. As shown in the figure, the keystone problem, the pincushion distortion and the color uniformity issue are well compensated. The diagonal FOV of the system is approximately 80 degrees, which is relatively superior to that of conventional AR systems. The real-world scene is slightly hazy due to the grain structure of the LDHOEs. Additionally, as presented in Fig. 4(c), the edge of the virtual image is slightly out of focus. An explanation for this phenomenon will be provided in the discussion session.

**Figure 5:**
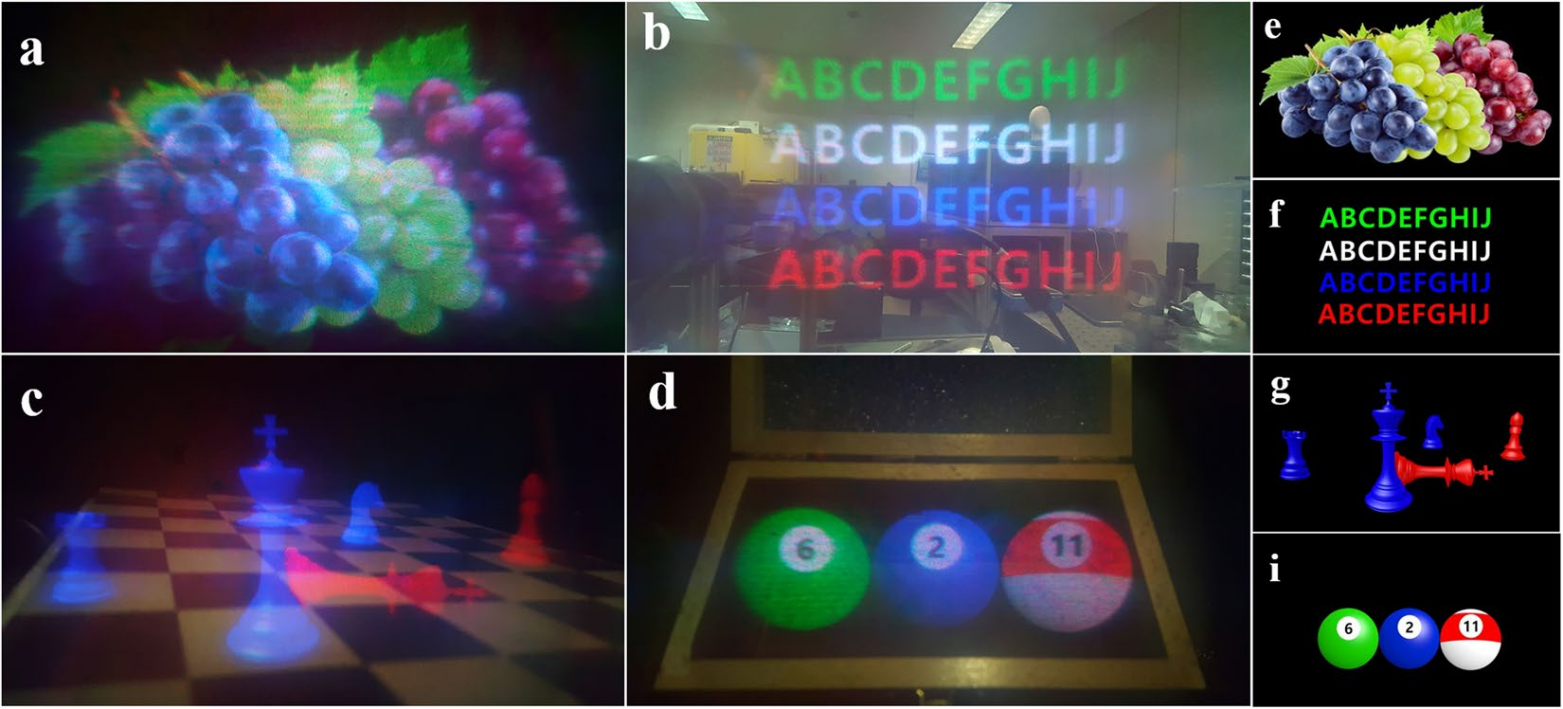
Experimental results. (**a**) A grapes image while the lights are turned off, (**b**) virtual letters floating in the real world, (**c**) virtual chess pieces on a real chess board, and (**d**) virtual billiard balls in a real wooden box. (**e**–**i**) present the target images.

Figure 5 represents the experimental results. Four different target images are employed. An image of grapes is used when the lights are turned off to show the exact quality of the system, as shown in Fig. 5(a,b) demonstrates virtual letters floated in the real world. In Fig. 5(c,d), virtual chess pieces are standing on a real chess board and virtual billiard balls are inside a real wooden box. As presented, the proposed system can offer wide-FOV images without chromatic aberration, and the real-world scene is simultaneously delivered without any distortion.

**Figure 6:**
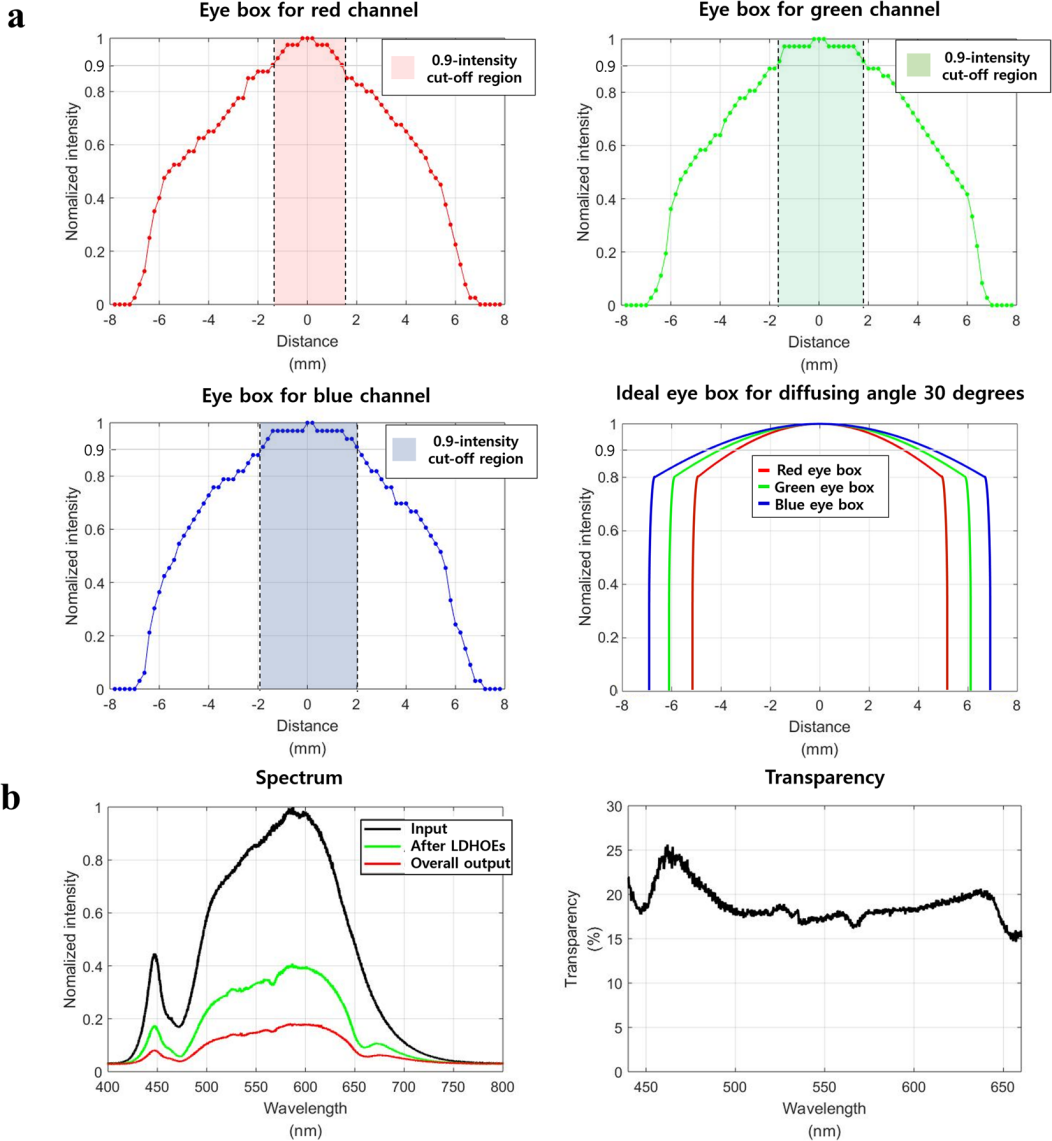
Analysis of the proposed system. (**a**) Eye box measurements for each color channel and the ideal eye box of the system when the diffusing angle is 30 degrees. (**b**) The spectrum of the input light and transmitted lights and the transparency of the proposed system achieved by the spectra.

To analyze the proposed AR near-eye display system, three additional experiments are conducted. First, the eye box of the system is measured using an illuminance meter. The results are presented in Fig. 6(a). The first three graphs show the eye box of the system for the red, green, and blue light sources. The colored region on each graph, which is regarded as the eye box, shows the 90% mark of the maximum intensity. The sizes of the measured eye boxes are 2.9 mm, 3.4 mm, and 3.8 mm for red, green and blue, respectively. Assuming that scattered light from the diffuser is uniformly distributed, the ideal sizes of the eye boxes, which are calculated using a ray matrix, are 3.21 mm, 3.87 mm, 4.37 mm. Considering that the light spreads with a Lambertian distribution, the measured values are reasonable. The fourth graph in Fig. 6(a) represents the ideal eye boxes for each color channel when the diffusing angle increases to 30 degrees. In this simulation, the Lambertian distribution is considered, and the intensity of the light in the maximum diffusing angle is supposed to be 80% of the intensity in the center. As presented in the figure, if the diffusing angle of the adopted diffuser is 30 degrees, approximately 10 mm of the eye box can be achieved. A detailed explanation is presented in the methods section. Second, the transparency of the system is analyzed.

As shown in the first graph in Fig. 6(b), the spectrum distribution of the input light and the output light is measured. The second graph in Fig. 6(b) represents the transparency of the system. In an ideal case, 50% of the light is lost after the light passes through the linear polarizer, and an additional 50% of the light is lost after the light passes through the PBLIC because there is a RHCP within the PBLIC. Thus, only 25% of the input light can penetrate the system even if the transparency of the layered LDHOEs is 100%. However, since the LDHOEs are not perfectly transparent to the light from the real world scene, the overall transparency is less than 25%, as presented in Fig. 6(b). Last, the degree of polarization retention is investigated after the light is diffused from the LDHOE. As mentioned above, the linearly polarized light from the projector is diffused by the LDHOEs and goes through the QWP and PBLIC. In this situation, if the polarization state of the light is not preserved after the diffusion, ghost images occur due to the other polarization components. The polarization states of the input light and the diffused light for each color channel are measured using a polarimeter. As a result, the ellipticity difference between the input light and the diffused light is 0.033 degrees, 0.012 degrees, and 0.02 degrees for red, green and blue light sources, respectively. Therefore, we concluded that ghost images from the polarization noise do not occur.

## Discussion

This paper proposes an AR near-eye display system using a novel combiner consisting of PB phase lenses. Since the combiner, named PBLIC, simultaneously has a small form factor and a high NA, it is advantageous to be adopted in AR near-eye displays. However, using PBLIC causes serious chromatic aberration due to the phase matching condition. To settle the chromatic aberration, a transparent screen composed of three layered LDHOEs is produced. Due to the diffusion in the different depths, the chromatic aberration can be compensated. In addition, adopting LDHOEs can achieve a relatively wide FOV compared to the system with conventional DHOEs because this system can handle the vignetting problem. Under the same conditions, the AR system with conventional DHOEs can provide an FOV of approximately 58 degrees, which is determined by the NA value of PBLIC, while the proposed method with LDHOEs presents an FOV of 80 degrees. However, adopting three layered LDHOEs may not be the ultimate solution to build the AR system using PB phase lenses. Stacking LDHOEs not only increases the overall form factor but also lowers the transparency. Hence, our system can be a practical solution in the present situation, but a better solution dealing with the chromatic aberration may be proposed in the future. For instance, achromatic metalens is a representative example of the foothold to develop the AR display using PB phase lenses^29^.

As shown in Fig. 4(b), the edge of the virtual image is slightly out of focus. This phenomenon was analyzed in the previous research conducted in our research group^19^. According to this analysis, the phase matching condition in Eq. (1) can be altered as a function of the radial distance from the center of the axis. In other words, the focal length of the light is not only affected by the wavelength itself but is also affected by the radial distance from the center of the axis. Therefore, if the virtual image is provided with an excessively wide FOV, the edges of the image can be out of focus while the center of the image is well focused. We think that adopting a holographic display has the potential to deal with this issue. To be specific, if point spread functions (PSFs) of each part within the FOV are acquired using a CCD camera, it is possible to determine which optical aberrations occur at each point. This information can be used to derive the appropriate digital hologram, which can compensate for the nonuniform focus issue and other optical aberrations.

In summary, the proposed system provides a wide FOV compared to other previous AR techniques using large AR combiners, such as prisms or freeform optics^2–5^. Additionally, 4 mm of eyebox is achieved and the chromatic aberration is well compensated by adopting LDHOEs. However, since the system adopts three-layer LDHOEs and PBLIC simultaneously, the transparency is restricted to 20~25% which is relatively less than other AR techniques using HOEs^7–13^, and the system cannot provide a focus cue. To deal with the low transparency issue and to provide a focus cue simultaneously, the holographic display concept can be adopted. In this case, the transparency can be improved by replacing the LDHOEs with a single mirror-HOE, which can also reduce the overall form factor. The most important thing is generating appropriate holograms for each color channel. Since the focal length of PBLIC varies with the input wavelength, the process of deriving holograms for each wavelength will be different. Hence, the system should adopt three independent SLMs that are operated with three different light sources. Ultimately, the observer would perceive achromatic and focus on cue-generating 3D images with a clear real-world scene.

## Methods

### Recording LDHOEs and the performance of the proposed system

**Figure 7:**
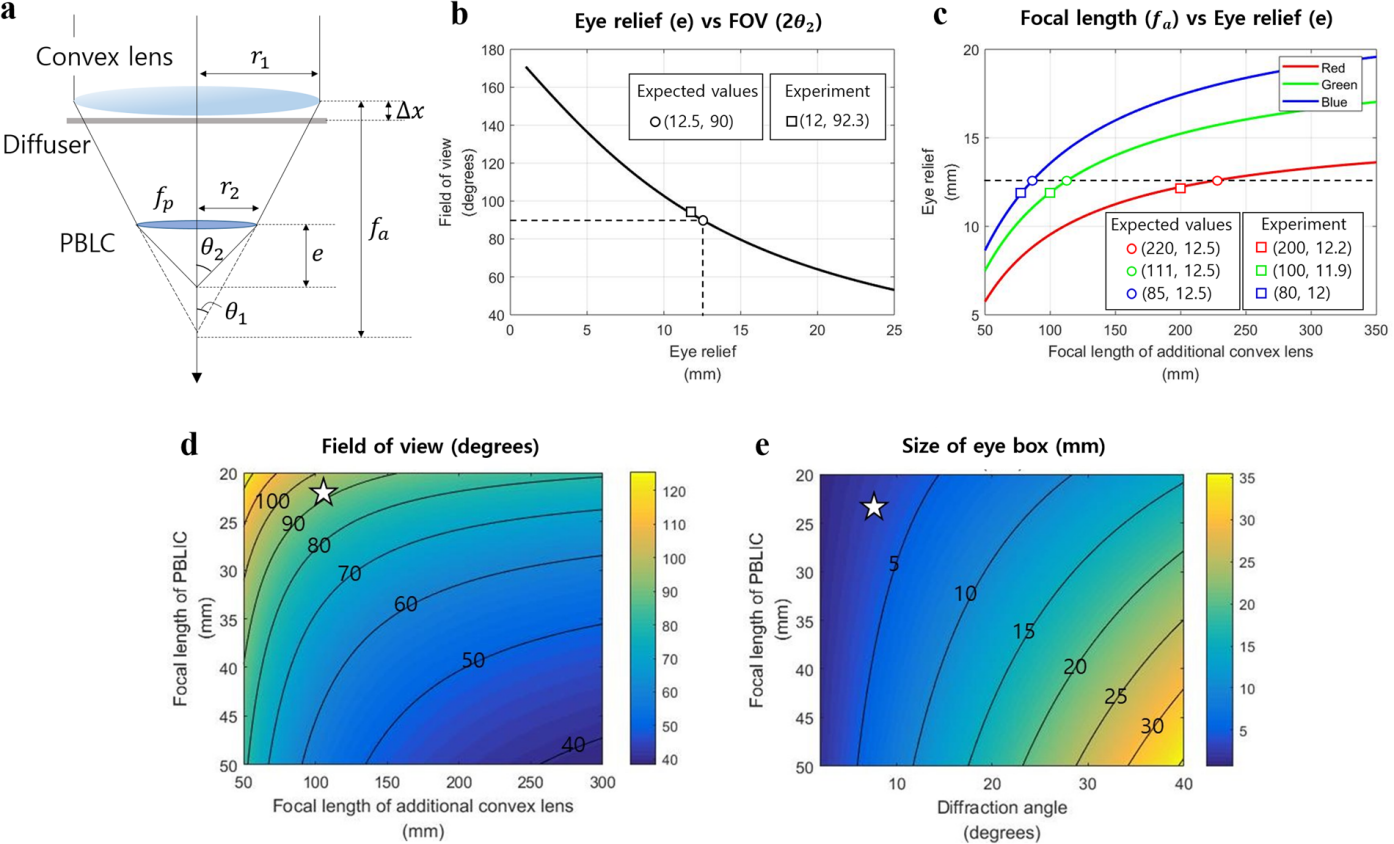
Relationships between system parameters and the ideal performance of the system. (**a**) The schematic diagram of system parameters. The relationship between (**b**) the eye relief distance and the FOV, and (**c**) the focal length of the additional convex lens and the eye relief distance. (**d**) Field of view of the system with various focal lengths of the PBLIC and the additional convex lens in the recording process. (**e**) Size of the eye box when varying the focal length of the PBLIC and the diffraction angles of LDHOEs.

In the LDHOE recording process, red (Cobolt, Flamenco wavelength 660 nm), green (Cobolt, Samba wavelength 532 nm), and blue (Cobolt, Twist wavelength 457 nm) lasers and a photopolymer (Coverstro, Bayfol HX TP star thickness 24 µm) are adopted. As demonstrated in Fig. 2(b), a convex lens is added in the conventional DHOE recording setup to manufacture LDHOE. Since the gaps between the PBLIC and each LDHOE are different, the focal length of the adopted additional convex lens should also be different to generate an identical eye relief distance. The eye relief distance is defined as the gap between PBLIC and the position where the size of the eye box is at its maximum. The target FOV of the proposed system is 90 degrees. To achieve the target FOV, the appropriate lens focal lengths for each color channel are calculated based on ray optics. Figure 7(a) is a schematic diagram of the system parameters. Under the paraxial approximation, the relationship of each parameter is as follows:

2 $${\theta }_{2}={\tan }^{-1}(\frac{{r}_{1}}{e})=\frac{{r}_{2}}{{f}_{p}}+{\theta }_{1}=\frac{{r}_{2}}{{f}_{p}}+{\tan }^{-1}(\frac{{r}_{1}}{{\rm{\Delta }}x+{f}_{a}}),$$

where *θ*_2_, *e*, *f*_*p*_, *f*_*a*_, and $\triangle x$ represent half the FOV, the eye relief distance, the focal length of the PBLIC, the focal length of the convex lens, and the gap between the convex lens and the diffuser, respectively. The radii of the convex lens (*r*_1_) and PBLIC (*r*_2_) are 25.4 mm and 12.5 mm, respectively. Figure 7(b) shows the relationship between the eye relief distance and the FOV. As mentioned above, the target FOV is 90 degrees, and the eye relief distance corresponding to this FOV is 12.5 mm. According to Fig. 7(c), to achieve an eye relief distance of 12.5 mm, the focal lengths of the convex lenses should be 220 mm, 111 mm, and 85 mm for the red, green, and blue light sources, respectively. The easily available convex lenses with focal lengths of 200 mm, 100 mm, and 80 mm are selected for the recording process. For these lenses, a simulation value of the eye relief distance is determined to be 12 mm, and the corresponding FOV becomes 92.3 degrees.

However, as presented in Fig. 4(b), the FOV measured in the experiments is approximately 10% less than the simulated value. This error occurs because the actual LDHOE screen size is smaller than the screen in the simulation. In the simulation, the gap between the convex lens and the diffuser ($\triangle x$) is assumed to be negligible, meaning that the screen size is 50.8 mm. However, during the recording process, the gap between the diffuser and the convex lens cannot be zero due to the thickness of the convex lens. The actual size of the screen is measured to be 32 mm, and the corresponding ideal FOV for this case is approximately 85 degrees. We expect that the FOV can be enhanced under improved recording conditions. Figure 7(d) shows the ideal FOV of the proposed system when varying the focal length of the additional convex lens and the focal length of the PBLIC. Figure 7(e) represents the size of the eye box with various LDHOE diffraction angles and the focal length of the PBLIC. The star-shaped mark represents the performance for the green light source in our system.

### Eye box, transparency, and polarization efficiency measurements

To measure the eye box of the proposed system, the luminance distribution at an eye relief distance of 12 mm is measured using a luminance meter (Konika Minolta, CA-210). The luminance distributions are measured by moving a luminance meter at an interval of 0.2 mm, as shown in Fig. 5(a). The transparency of the system is measured using a spectrometer (Oceanoptics, USB4000-VIS-NIR-ES). The spectrum of the flashlight is measured as the input source. Then, the spectra of the transmitted light after LDHOEs and PBLIC are measured individually, as demonstrated in Fig. 5(b). Finally, the polarization states are measured using a polarimeter (General photonics, POD-201). The polarization state of the linearly polarized input light sources are measured, and the polarization states of the output light diffused from LDHOEs are measured.
